# A review on high-resolution CMOS delay lines: towards sub-picosecond jitter performance

**DOI:** 10.1186/s40064-016-2090-z

**Published:** 2016-04-12

**Authors:** Bilal I. Abdulrazzaq, Izhal Abdul Halin, Shoji Kawahito, Roslina M. Sidek, Suhaidi Shafie, Nurul Amziah Md. Yunus

**Affiliations:** Department of Electrical and Electronic Engineering, Universiti Putra Malaysia (UPM), 43400 Serdang, Selangor Malaysia; Department of Electronic and Communications Engineering, Al-Nahrain University, Al-Jadriya Complex, Baghdad, 10070 Iraq; Imaging Devices Laboratory, Research Institute of Electronics, Shizuoka University, 3-5-1 Johoku, Nakaku, Hamamatsu, Shizuoka 432-8011 Japan

**Keywords:** Delay line, Delay resolution, Delay range, Jitter, Delay element, Linearity, Delay-locked loop (DLL)

## Abstract

A review on CMOS delay lines with a focus on the most frequently used techniques for high-resolution delay step is presented. The primary types, specifications, delay circuits, and operating principles are presented. The delay circuits reported in this paper are used for delaying digital inputs and clock signals. The most common analog and digitally-controlled delay elements topologies are presented, focusing on the main delay-tuning strategies. IC variables, namely, process, supply voltage, temperature, and noise sources that affect delay resolution through timing jitter are discussed. The design specifications of these delay elements are also discussed and compared for the common delay line circuits. As a result, the main findings of this paper are highlighting and discussing the followings: the most efficient high-resolution delay line techniques, the trade-off challenge found between CMOS delay lines designed using either analog or digitally-controlled delay elements, the trade-off challenge between delay resolution and delay range and the proposed solutions for this challenge, and how CMOS technology scaling can affect the performance of CMOS delay lines. Moreover, the current trends and efforts used in order to generate output delayed signal with low jitter in the sub-picosecond range are presented.

## Background

Delay lines are devices that introduce time delay to signals by a pre-determined time constant. They are characterized by their delay step, jitter performance and delay range. The delay step is a measure of the finest incremental time step a delay line can produce, while the delay range is the maximum time a signal can be delayed. On the other hand, jitter is the time uncertainty in an output delayed signal and directly affects the smallest delay step (Abas et al. 2007a; Alahmadi 2013; Kalisz 2004; Napolitano et al. 2010; Xanthopoulos 2009; Otsuji and Narumi 1991). Delay lines play a substantial role in many sub-systems of time interval measurement (TIM) circuits such as time-to-digital converters (TDCs) and digital-to-time converters (DTCs) for digitization of short time intervals (Rahkonen and Kostamovaara 1993; Andreani et al. 1999). Delay lines also find applications in range imaging where a delayed light pulse is required to capture 3-dimensional range images (Charbon et al. 2013). In the computer industry, a digitally tapped-delay line (TDL) is used to move, delay, and store data at precise time windows for data synchronization purposes (Weste and Harris 2011a). Moreover, CMOS delay lines are used in the applications of clock distribution and clock-data recovery (CDR) to satisfy the growing needs for precise clock deskew, and in accurate pulse-edge placement control for testing and debugging the dynamic behavior of high-speed and high-performance digital VLSI circuits (Sakamoto et al. 1989; Maymandi-Nejad and Sachdev 2005). CMOS delay lines are also used in on-chip time measurements and the synchronization of a CPU with its interfaces (Andreani et al. 1999; Abas et al. 2007a).

There are two types of delay lines available in industry, which are based on optical technology and electronic technology. Optical delay lines offer the highest-resolution delay step, truly in the sub-picosecond range and with exceptionally linear increments. A signal is delayed by adjusting an air gap distance between an input and output fiber optic transceiver. The greater the distance a light signal travels between these two points, the longer the time delay of the output signal. However, optical delay lines offer limited delay range, which is in the order of only a few 100 ps. When an application calls for exceptionally long delays, several optical delay lines can be cascaded to extend the range with no loss in resolution and linearity (Melloni et al. 2010). However, this is achieved at the expense of system complexity since optical delay lines use fiber optic cables which make for a costly, bulky and fragile setup (Hashimoto et al. 2008). On the other hand, CMOS delay lines offer reduced system complexity and cost (Hashimoto et al. 2008; Melloni et al. 2010).

There are two main issues with conventional CMOS delay lines. The first issue is the jitter performance which is in the range of several picoseconds (Klepacki et al. 2014; Xanthopoulos 2009). Although the jitter performance is not as fine as that of optical-based delay lines, extensive work to produce sub-picosecond jitter performance CMOS delay lines is actively undertaken by many parties due to the fact that IC-based delay lines are robust in terms of system integration and cost reduction when compared to their optical counterpart. The second issue is in realizing a long delay range that is linear with high-resolution delay steps simultaneously (Xanthopoulos 2009). Fine-resolution CMOS delay lines cannot simply be cascaded like optical delay lines because delay increments are non-linear mainly due to the complex nature of the parasitic capacitance network in the delay elements of the delay line. The cascading methodology also leads to a complex PCB implementation. Thus, a single chip solution should be developed to overcome these shortcomings.

This paper focuses on state of the art research on high-resolution and high jitter performance CMOS delay lines. The performance parameters of delay lines fabricated using the most recent CMOS technology were reported by (Schidl et al. 2012) using 90 nm. The jitter performance and the delay range reported for this 90 nm-delay line are 0.6 ps RMS and 155 ps, respectively, using the analog-tunable SCI-based delay line technique. Although the latest delay line found was fabricated using 0.13 µm (Han et al. 2016), material on implemented delay lines using the most recent CMOS technology is reported for 90 nm (Schidl et al. 2012) in this review paper. However, excellent jitter performance and delay resolution using all technology nodes are discussed in detail in this paper. Breakthrough delay circuit architectures that produce either fine or coarse delays are presented early in this paper focusing on their functionality and delay characterization. Analog and digitally-controlled delay elements are then presented with a focus on the most common delay-tuning strategies utilized by CMOS delay lines designers. The effects of CMOS technology scaling and PVT variations on CMOS delay lines performance are also presented. Subsequently, a topic on noise and timing jitter sources of delay lines is presented. Common techniques for generating sub-gate delay resolution are summarized in the last section, and the collection of delay circuits presented is also summarized and compared to highlight the trade-off between maximum delay and delay resolution. Likewise, other performance metrics, like jitter, linearity, robustness to PVT variations, power consumption and occupied area are also compared and discussed in the last section for the common delay line circuits.

## CMOS delay line circuit architecture

**Fig. 1:**
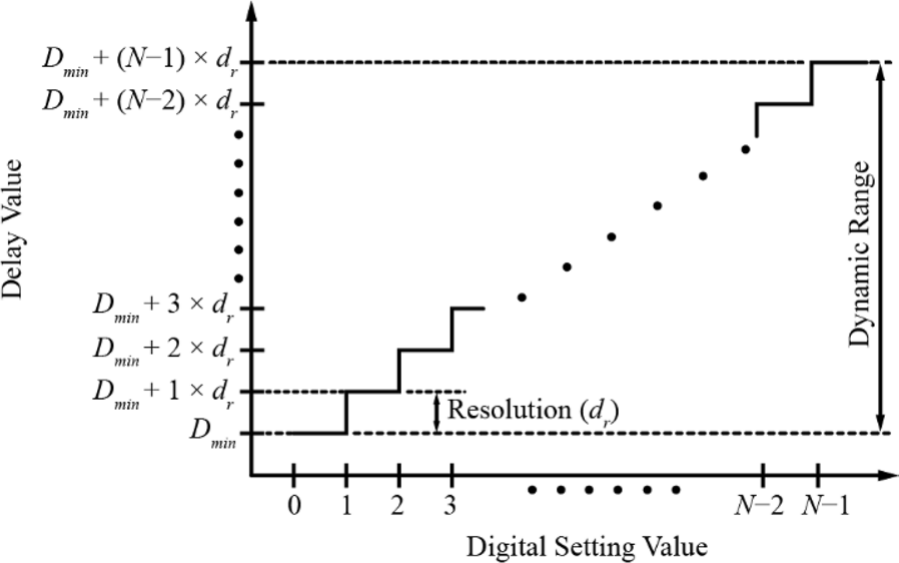
DCDL’s transfer function (Xanthopoulos 2009)

CMOS delay lines come in a variety of architectures. Each architecture is attributed to how a controlled delay is produced by the circuit. There are two methods in controlling the delay, which are through a digital word whose value directly maps to the desired delay or through an analog signal (Maymandi-Nejad and Sachdev 2005). The latter is usually used for sub-picosecond to picosecond delay control (Schidl et al. 2012). Figure 1 shows the transfer function of a CMOS digitally-controlled delay line (DCDL).

The x-axis shows the decimal equivalent of a desired delay value while the y-axis shows the corresponding output time delay value, *T*_*d*_, and is given by:

1 $$T_{d} = D_{\hbox{min} } + (N - 1) \times d_{r}$$

where *d*_*r*_, *D*_min_, and *N* are the delay resolution (smallest achievable delay step), minimum delay (delay value at setting 0), and the number of programmable delay bits, respectively. For example, according to Eq. (1), a time delay of 3 ns is produced on an 8-bit delay line, when *D*_*min*_ = 0.55 ns and *d*_*r*_ = 0.35 ns.

Contemporary CMOS delay lines reveal a trade-off between delay range and resolution, thus several studies on enhancing both of these parameters for applications in circuit synchronization and clocking have been undertaken (Xanthopoulos 2009; Rahkonen and Kostamovaara 1993). This is achieved through a multi-stage architecture. For example, a wide delay stage is designed using a counter that counts clock periods to produce a long delay period with coarse delay increments. The fine delay stage is based on an interpolator circuit that subdivides and resolves the fractional parts of the clock period from the coarse delay stage into smaller and finer time windows. This allows finer delay steps within the coarsely delayed signal. Although it may seem interesting in concept, matching of the delay elements limits the resolution and the maximum length of the delay line (Rahkonen and Kostamovaara 1993; Xanthopoulos 2009; Kalisz 2004; Nutt 1968). It has been shown that an increase in delay line length to obtain longer delays will increase timing jitter of the output delayed signal by the square root of the delay line length (Klepacki et al. 2014; Nuyts et al. 2014; Henzler 2010a).

Although there are many different circuits that can be used to design delay lines, their architecture can be classified into two which are the tapped and single-output delay line architectures. The circuit architecture differs as follows:

1. Tapped-delay line (TDL) architecture:

This architecture, also called fixed-delay line, makes use of *N* identical delay elements. Each delay element is connected in series. The output is tapped out at each stage using a switch. Depending on the required delay step, the delay elements may be designed using static logic gates or flip-flops (Rahkonen and Kostamovaara 1993; Abas et al. 2007a; Alahmadi 2013). The finest delay step is limited to the propagation delay of a single delay element, depending on the speed of the CMOS technology used (Nuyts et al. 2013; Henzler 2010b). The delay range is approximately equal to the product of the finest delay step by the number of delay stages (Alahmadi 2013).

**Fig. 2:**
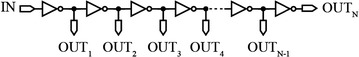
Inverter Chain delay line (Nuyts et al. 2014)

Figure 2 shows an example of how static logic gates (inverters) are used as delay elements in a TDL. It is designed using *N* inverters connected in series where two adjacent inverters form a delay element. Thus, its delay step is equal to the propagation delay of two inverters and is determined by the equivalent drive resistance and the output load capacitance of the inverter.

A non-inverted output is tapped from the even numbered outputs $({\text{OUT}}_{2} ,{\text{OUT}}_{4} , \ldots ,{\text{OUT}}_{\text{N}} )$ (Nuyts et al. 2014; Mahapatra et al. 2000; Ihrig et al. 2009).

**Fig. 3:**
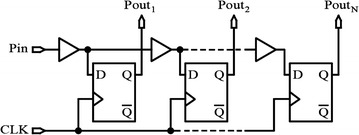
A tapped (fixed) delay line (Abas et al. 2007a)

Figure 3 shows another implementation of a fixed delay line using D-flip-flops and buffers as the delay elements. The outputs P_out1_–P_outN_ are delayed from the input, P_in_, by a fixed amount of delay, where the delay of P_outN−1_ is less than that of P_outN_ by approximately the propagation delay of the buffer and D-flip-flop operated at a known clock speed (Abas et al. 2007a). It is concluded that the tapped delay line architecture has only a single input and multiple outputs from multiple delay elements that are selected based on the delay desired.

Since the propagation delay of logic gates-based delay elements plays a crucial role in determining the delay resolution and range of the TDL, it is important to know the parameters that have a first-order impact on the delay of logic gates. This can be illustrated through the following general CMOS gate delay equation (Segura et al. 2006):

2 $$\tau_{D} = \frac{{2LC_{L} V_{DD} }}{{W\mu C_{ox} (V_{DD} - V_{TH} )^{\alpha } }} .$$

where *L*, *W*, *C*_*L*_, *V*_*DD*_, *µ*, *C*_*ox*_, *V*_*TH*_, and *α* are the transistor channel length, channel width, load capacitance, supply voltage, carrier mobility, gate oxide capacitance, threshold voltage, and a technology parameter used to express the carrier-velocity saturation effect, respectively. *α* has a value ranging between 1 and 2 for short and long channel devices, respectively.

In relations to the shrinking of gate feature-size as MOS technology advances to deep sub-micron (DSM) and ultra-DSM technologies, the gate delay becomes smaller. This is strongly correlated to the propagation delay of logic gates in any given CMOS process. Although choosing smaller feature-size transistors in DSM or UDSM technology for a high-resolution TDL design seems attractive (Zhang and Kaneko 2015), one must not forget the effects of interconnect resistance, negative bias temperature instability (NBTI), random doping fluctuations, gate-oxide tunneling, PVT variations and short channel effect which become more and more significant (Jiang 2011; Segura et al. 2006; Ghahroodi 2014). These effects ultimately contribute to excessive timing jitter which should be minimized. Besides that, utilizing wider transistors is not useful in enhancing the delay resolution as the gate capacitance of logic gates is increased simultaneously (Zhang and Kaneko 2015; Nuyts et al. 2014).

2. Single-output delay line architecture:

Unlike the tapped delay line, a single-output delay line, as its name suggests, has only one output. The ability to adjust the desired output signal’s time delay is done either through an analog or digital signal depending on the type of delay element used. Usually, if a current-starved delay element (Maymandi-Nejad and Sachdev 2003) or shunt-capacitor delay element (Andreani et al. 1999) is used, a digital input word is required to change the delay. On the other hand, if an analog differential buffer (Nuyts et al. 2014; Maneatis 1996) or a MOS diode-based delay element (Markovic et al. 2013) is used, an analog signal is required. More on these types of delay circuits and other types will be discussed in the next two sections.

**Fig. 4:**
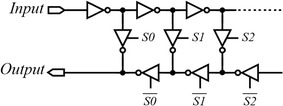
A network of inverters-based delay line (Abas et al. 2007a)

Figure 4 shows an example of a single-output delay line. This type of architecture changes its delay by including and excluding delay elements in its signal path. The delay elements are made from static logic gates, thus having a propagation delay in the order of several nanoseconds (Mahapatra et al. 2000).

Tri-state inverters controlled by complementary signals *S*0, *S*1 and *S*2 are used to serially connect/disconnect an even number of inverters in the signal path. For example, a delay equal to six inverter delays is generated at the *Output* when *S*0 and *S*1 are ‘0’ and *S*2 is ‘1’. The delay step for this type is limited to two inverter delays (Abas et al. 2007a).

**Fig. 5:**
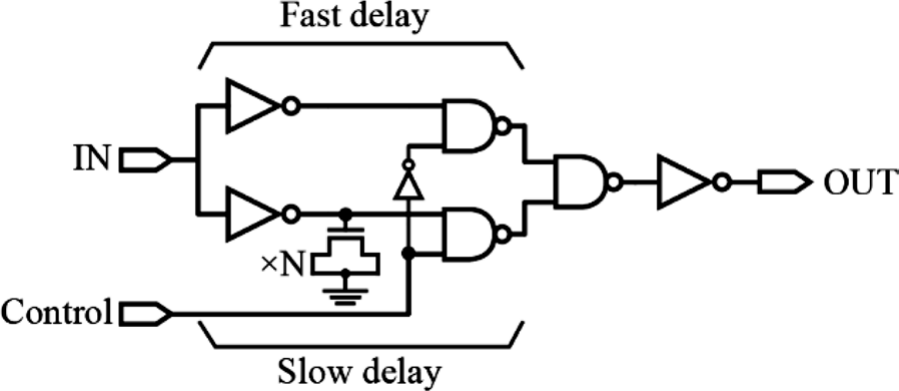
A delay line based on delay differences with sub-gate delay resolution (Guang-Kaai et al. 2000)

A delay line, using logic gates, that operates based on the delay difference between two delay paths is shown in Fig. 5. The input pulse, fed to the input IN, propagates through two different signal paths that have slightly different delay times (fast delay and slow delay). The difference in delay is due to the addition of a string of N number of MOS capacitors connected at the slow delay path. The signal labeled as Control is used for selecting the signal path. Hence, a sub-gate resolution delayed output is obtained, where the delay is equal to the difference of propagation delay time between the upper and lower signal paths (Xanthopoulos 2009; Guang-Kaai et al. 2000). This delay line circuit technique is sometimes referred to as vernier delay line (VDL).

It can be summarized that the delay resolution and delay range of the TDL and some of the single-output delay line architectures mainly depend on the propagation delay and thus on the CMOS technology used. Although choosing smaller gate-length and/or wider transistors in DSM/UDSM technologies seems attractive to enhance the delay resolution, many negative effects become more significant and can contribute to excessive jitter.

## Analog-tunable delay elements

A delay element is a circuit that is fundamental to any delay line. It is responsible for generating an output signal waveform almost identical to the input but delayed by a pre-selected amount of time. Aside from delay lines, delay elements also find wide use in many digital and mixed-mode signal circuits including DLLs and PLLs for phase modulation, asynchronous or self-timed circuits, multi-clock domain synchronization, microprocessors and memory circuits, and local timing generators (Maymandi-Nejad and Sachdev 2005; Ihrig et al. 2009; Mahapatra et al. 2000).

Delay elements can be categorized into two types, passive and active delay elements. Passive delay elements are constructed using passive devices such as resistors, inductors, and capacitors. They are less sensitive to environmental variations, have better linearity, cause less distortion to the output signal, and have a wider bandwidth with better accuracy (Mota and Christiansen 1999; Analui and Hajimiri 2003; Adabi and Niknejad 2008). Alternatively, active delay elements are circuits whose main elements are active components such as transistors and diodes. They are programmable and offer finer delay steps. Active delay elements can further be classified into coarse and fine delay elements. Coarse delay elements provide fixed, quantized and longer time delays and are always used to implement long-range delay lines. On the other hand, fine delay elements produce small and precise delay steps by means of an analog control voltage or current and are suitable for designing sub-picosecond step delay lines (Mahapatra et al. 2002; Maymandi-Nejad and Sachdev 2005; Adabi and Niknejad 2008). Equation (3) expresses the relationship between an analog-controlling current and the delay time of an active delay element (Eto et al. 2000).

3 $$\frac{{I_{0} -\Delta I}}{{I_{0} }} = \frac{{\tau_{D} }}{{\tau_{D} +\Delta \tau_{D} }}$$

*I*_0_, ∆*I*, *τ*_*D*_, and ∆*τ*_*D*_ are the controlling current of the delay element, change in controlling current, delay time of the delay element, and the change in delay time, respectively. Equation (3) shows that when *I*_0_ is decreased by ∆*I*, the total delay time will be *τ*_*D*_ + ∆*τ*_*D*_, which is an increase in delay time (Eto et al. 2000). From Eq. (3), it is seen that the controlling current dictates the delay because it charges and discharges the output capacitance of a delay element.

The delay of CMOS delay elements can be tuned/varied by varying the RC time constant of the delay element via changing the effective ON resistance or effective capacitance (Yang 2003; Nuyts et al. 2014). For logic gates-based delay elements, this can be achieved through two strategies. The first strategy is via changing the drive strength of a logic gate driving both a capacitor and the input of a second logic gate. The second strategy is through adding a variable load located at the internal node between two successive logic gates which are forming a clock buffer (Nuyts et al. 2014; Schidl et al. 2012).

The drive strength can be changed through two main methods which are changing the power supply voltage, also called supply modulation (Nuyts et al. 2014; Klepacki et al. 2014; Yang 2003), and current-starving (Schidl et al. 2012; Nuyts et al. 2014; Klepacki et al. 2014). The current-starving method can be implemented using many techniques, but the main are: adding delay-controlling MOS transistors of controlled aspect ratio which act as adjustable current sources at the pull down and/or pull up networks (Maymandi-Nejad and Sachdev 2005; Klepacki et al. 2014; Henzler 2010b; Rahkonen and Kostamovaara 1993), connecting additional delay-controlling MOS transistors at the output of logic gates as in the case of a transmission gate placed at the output of a logic gate (Nuyts et al. 2014; Mahapatra et al. 2002), employing a neuron-MOS mechanism which is based on an nMOS transistor with a gate electrode electrically floating (Zhang and Kaneko 2015; Shibata and Ohmi 1992), and employing an RC-based differentiator to drive the pMOS transistor of a CMOS inverter (El Mourabit et al. 2012).

**Fig. 6:**
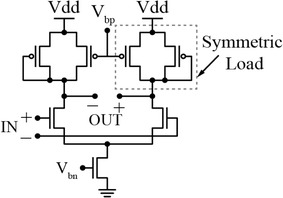
Analog differential buffer delay element (Maneatis 1996)

These delay-controlling/tuning techniques change the rate at which the output effective capacitance is charged/discharged. On the other hand, adding a variable load or sometimes referred to as load-increasing strategy (Zhang and Kaneko 2015) is implemented either by adding explicit tunable output capacitance(s) (Andreani et al. 1999; Yang 2003; Schidl et al. 2012) or by controlling the charging/discharging current of the node of MOS-diode at the internal node of a logic-gates-based buffer (Markovic et al. 2013; Klepacki et al. 2014). For analog delay elements, such as the one illustrated in Fig. 6, the delay is varied by varying the gate voltage V_bp_ and the biasing current source.

Five examples of analog-controlled/tunable delay elements are presented in this section.

The analog differential buffer delay element, shown in Fig. 6, has been used to attain sub-gate delay resolution (Nuyts et al. 2014). It has improved spectral purity and high immunity to common mode noise (Jia 2005). It consists of a source-coupled differential pair with resistive active symmetric loads and a biasing tail current source. The load is changed by varying V_bp_ which in turn varies the drain current of the two input transistors, thus varying its speed and the output delay of the circuit (Maneatis 1996).

Another delay circuit which has also been utilized to produce delay steps with sub-gate delay resolution is the delay-locked loop (DLL) (Xanthopoulos 2009; Yang 2003; Eto et al. 2000). DLLs are unconditionally stable time-delay circuits and capable of generating delayed output signals that have a precise phase relationship with an input reference signal by employing phase interpolation (Xanthopoulos 2009; Yang 2003). The main advantage of using the DLL is that the generated time delay is exceptionally stable against PVT variations and noise sources compared to other types of delay elements (Markovic et al. 2013; Rahkonen and Kostamovaara 1993) as the jitter performance of DLLs has been quoted in the picosecond range (Jaehyouk et al. 2011; Helal et al. 2008).

**Fig. 7:**
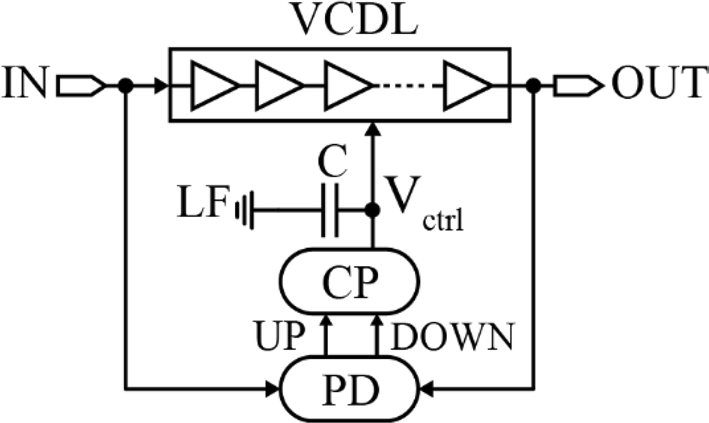
Analog delay-locked loop architecture (Jovanovic et al. 2005)

In general, analog DLLs are capable of generating a high-resolution delay step (Jia 2005) with low jitter (Jia 2005; Yongsam et al. 2000; Hsiang-Hui and Shen-Iuan 2005). Moreover, they have higher power supply and substrate noise rejection (Jia 2005). However, they are affected at large by process variations (Kuo-Hsing and Yu-Lung 2007). An analog DLL circuit is shown in Fig. 7. It comprises of a phase detector (PD), a charge pump (CP), a loop filter (LF), and a voltage-controlled delay line (VCDL) (Kuo-Hsing and Yu-Lung 2007; Jia 2005). Referring to Fig. 7, the signal of the input reference clock propagates through the delay stages of the VCDL and hence, a unit phase shift is generated at every delay stage output. The phase of the delayed output signal is compared with that of the input clock signal by the PD. Depending on the phase difference; PD generates phase error information which is converted to a charge by the CP to tune the LF’s control voltage. Consequently, the time delay of each delay element is varied. Repeating this mechanism through the negative feedback closed-loop, the phase error is gradually minimized until it becomes zero. Meanwhile, the DLL locks indicating that a stable locking state is obtained, and the LF’s voltage is stabilized (Jia 2005; Xanthopoulos 2009).

DLLs are characterized by four performance metrics, namely, lock range, locking time, jitter performance, and static phase error (Cheng and Milor 2009). Lock range indicates the maximum and minimum delays of the VCDL and directly affects the DLL’s operating frequency range (Jia 2005). Lock range can be increased by including more delay elements in the VCDL, for example (Yeon-Jae et al. 2001; Yang 2003). The locking time refers to the time required for a DLL to reach a stable locking state from an initial state. Jitter is a measure of random fluctuation in output delay time about a fixed/desired value. Thus, jitter and delay resolution is closely related (Jia 2005; Otsuji and Narumi 1991). Finally, static phase error indicates the phase (delay) difference between the output delayed signal of the VCDL and the input signal to the DLL. Ideally, perfect matching of these two signals’ phases should be established after DLL’s locking state is achieved. Nonetheless, some static phase error is introduced because of the limited resolution of the PD and the CP. Static phase error is very sensitive to device speed and temperature as slow devices and high temperature result in slow switching of the transistors, thus contributing to large static phase error (Cheng and Milor 2009).

The DLL’s loop bandwidth plays a significant role in controlling the DLL’s performance metrics. For example, increasing DLL’s bandwidth leads to an improvement in the lock range and locking time. However, increasing the bandwidth can less effectively filter out high-frequency components of the VCDL’s phase noise, and it results in degradation of the jitter (Cheng and Milor 2009).

The delay step in DLLs can be defined as the finest change in the delay time of the output signal after a DLL’s output has been locked. The delay step is controlled by the CP voltage. To explain this, the analog control voltage signal from the CP is applied to the delay elements to control/tune the delay. This signal, applied to the gates of the delay-controlling transistors, precisely changes the current responsible for charging/discharging the output capacitance of the delay element. Consequently, and according to the current ratio relationship in Eq. (3) for fine delay control, the delay is varied precisely and a sub-gate delay resolution can be achieved.

On the other hand, the delay line’s maximum delay can be defined as the maximum achievable lock range of the DLL. Basically, in order to achieve a long delay range, the VCDL should produce long delays. There are essentially two techniques to design the VCDL for this objective. The first technique is by utilizing a large number of delay elements having a comparatively short unit delay. The drawbacks of this method are increased power consumption and area. The second method utilizes a smaller number of delay elements with a considerably long unit delay. However, this has the shortcoming of producing signals with slow-switching transition edges which are more prone to deteriorate jitter performance of the unit delay element (Jaehyouk et al. 2011; Moazedi et al. 2011). The jitter characteristic of the DLL heavily depends on that of the VCDL. The total VCDL’s jitter represented by the total timing error variance, $\sigma (\Delta t_{VCDL}^{2} ),$ is written as:

4 $$\sigma \left( {\Delta t_{VCDL}^{2} } \right) = \sigma \left( {\Delta d^{2} } \right) \cdot \frac{2N}{{2 - \frac{{I_{CP} K_{d} }}{{C_{L} }}}}$$

where *σ*(∆*d*^2^), *N*, *I*_*Cp*_, *K*_*d*_, *C*_*L*_ are the timing error variance of the unit delay element, number of delay elements in the VCDL, CP current, delay element gain, and capacitance of the LF’s capacitor, respectively (van de Beek et al. 2002). From Eq. (4), it is clear that by simply increasing the number of delay elements to increase delay range, the jitter performance is degraded.

When utilizing DLLs as delay lines, the generated jitter which is found at the output of DLLs comes from four different sources, which are the input reference clock, PD, CP, and the VCDL jitter (van de Beek et al. 2002). It should also be noted that there is a trade-off relating the DLL’s lock range and jitter. For example, increasing the DLL’s lock range (delay range) also increases the jitter (Jaehyouk et al. 2011).

**Fig. 8:**
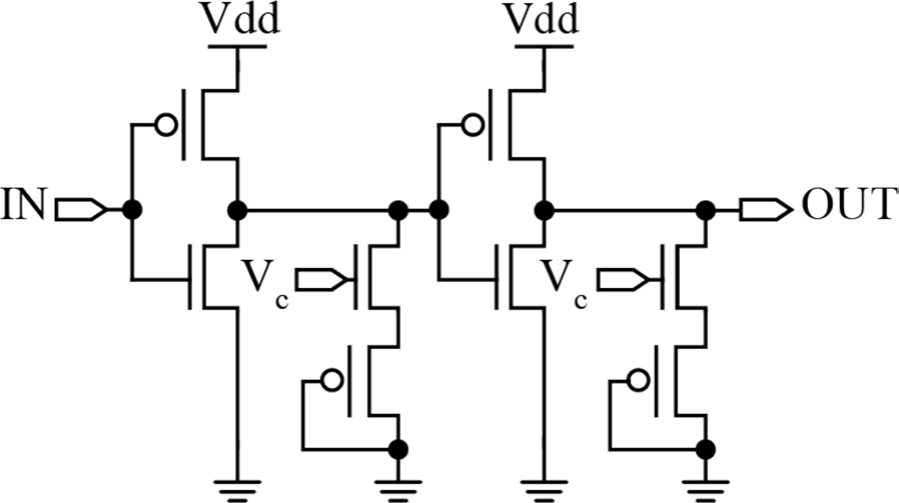
MOS diode-based delay element (Markovic et al. 2013)

Diode-connected transistors have also been used as delay elements. An implementation used for fine DLLs and interpolators in a Time-to-Digital Converter is shown in Fig. 8.

The output of an inverter is loaded with a diode-connected pMOS transistor. The diode capacitance charging/discharging current is controlled through gates V_c_, allowing the delay to be varied. V_c_ is an analog control signal provided by a CP of a DLL circuit (Markovic et al. 2013).

**Fig. 9:**
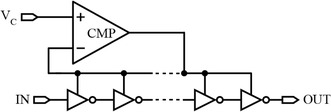
Tunable logic gate-based delay element based on supply modulation (Yang 2003)

Another type of delay elements in which the delay is varied by regulating the supply voltage is shown in Fig. 9. A control voltage, V_c_, is used to regulate/change the supply voltage. Accordingly, for variable supply voltages, the transistors of the logic gates are allowed to draw variable current values, therefore changing the rate at which the output effective capacitance is charged or discharged. This leads to a tunable delay for the delay element. However, one of the limitations of this technique is that it needs an adjustable analog voltage source capable of providing a considerable amount of current (Nuyts et al. 2014; Yang 2003). Another limitation is the highest achievable delay resolution which is not as fine as that of the other delay-controlling techniques reported in this paper (Moazedi et al. 2011).

**Fig. 10:**
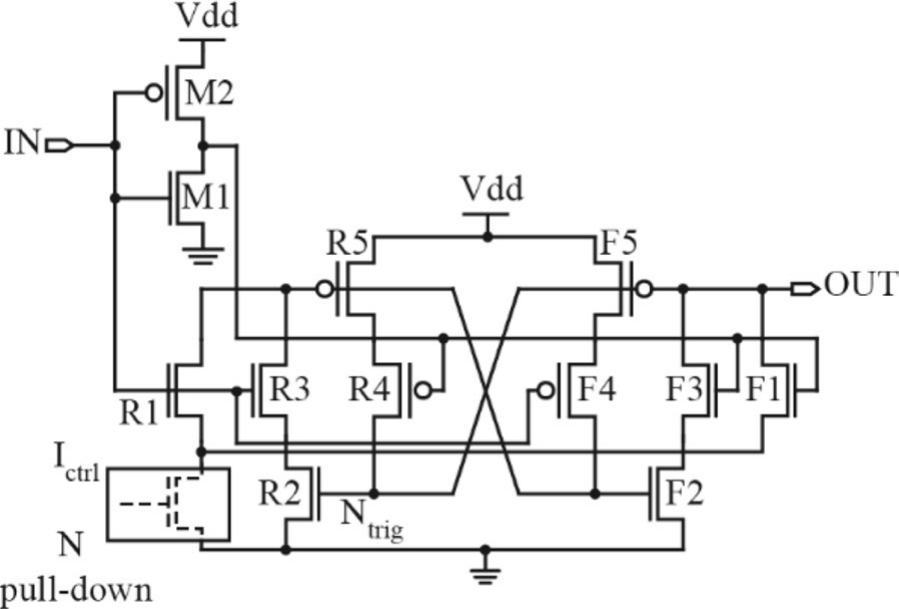
CMOS Thyristor-based delay element (Mahapatra et al. 2000)

The CMOS Thyristor delay element is shown in Fig. 10. It has a good robustness against environmental variations because this architecture is current-controlled rather than voltage-controlled (Junmou et al. 2004). It also produces long-range delays where the delay range is directly proportional to the lengths and the number of transistors in the *N* pull-down network (Kim et al. 1996; Mahapatra et al. 2000).

It is summarized that the delay elements are the fundamental building blocks for the CMOS delay lines. For delay-tuning functions, there are two main strategies which are changing the drive strength of the delay element and load-increasing strategy. Analog-tunable delay elements are the recommended choice when high-resolution delay step, low jitter, good intrinsic calibration for PVT variations and good stability are considered together in the design. Apart from analog-controlled delay elements, digitally-controlled delay elements are also being developed as they offer robustness and simplicity when it comes to design and delay control. The next section discusses these circuits in detail.

## Digitally-controlled delay elements

These types of delay elements are designed using logic gates. Delay is controlled using a digital word, where ideally a linear binary increment of the word corresponds to a linear increment of output delay. There are four main types of picosecond-resolution delay elements which are the shunt-capacitor inverter (SCI), current-starved inverter (CSI), inverter matrix, and the differential delay cell (DDC) (Abas et al. 2007b; Alahmadi 2013).

**Fig. 11:**
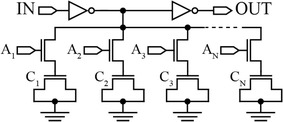
An SCI-based delay element (Abas et al. 2007a)

Figure 11 shows an SCI delay element. A MOS capacitor network is connected as the load to the input inverter via nMOS switches. By selecting the capacitors through control pins A_1_ through A_N_, the load can be varied, therefore changing the rise and fall times of the inverted input signal. This signal then passes through the output inverter which inverts the signal back to resemble the input but with added fine delay (Abas et al. 2007a, b). In another implementation of this type of delay elements in 0.35 μm CMOS technology, a 1.43 ps delay resolution has been achieved with a delay range of 40 ps (Pao-Lung et al. 2005).

**Fig. 12:**
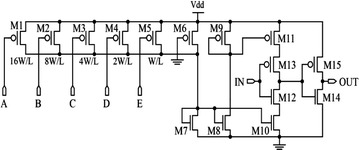
A CSI-based delay element (Maymandi-Nejad and Sachdev 2005)

The CSI architecture is shown in Fig. 12. It comprises of a pair of inverters (M12–M15), a set of current source (M1–M5), and two output current mirrors (M7–M11). The current mirrors are used to separate the set of current source from the inverters-based buffer stage. Thus, the non-monotonic delay behavior problem can be obviated by avoiding the junction capacitances on the dis/charging paths (Zhang and Kaneko 2015; Maymandi-Nejad and Sachdev 2005). The current through load transistor (M6) which is connected to the current mirrors can be changed via parallel connected transistors, M1–M5. These transistors also act as binary-weighted resistors and signals A, B, C, D, and E allow control of their total resistance, hence modifying the currents through M10 and M11, resulting in a change in the speed and response of the inverters. This architecture has proven to produce a delay step of 2 ps with a delay range of 320 ps using 0.18 µm CMOS technology (Maymandi-Nejad and Sachdev 2005).

Although the idea of using binary weighted transistors to precisely control delay step sounds attractive, linear step increments are only possible by fine-tuning the size of transistors M1 through M5 as interaction of parasitic capacitance of these transistors affects the binary weights, causing non-linear delay steps.

**Fig. 13:**
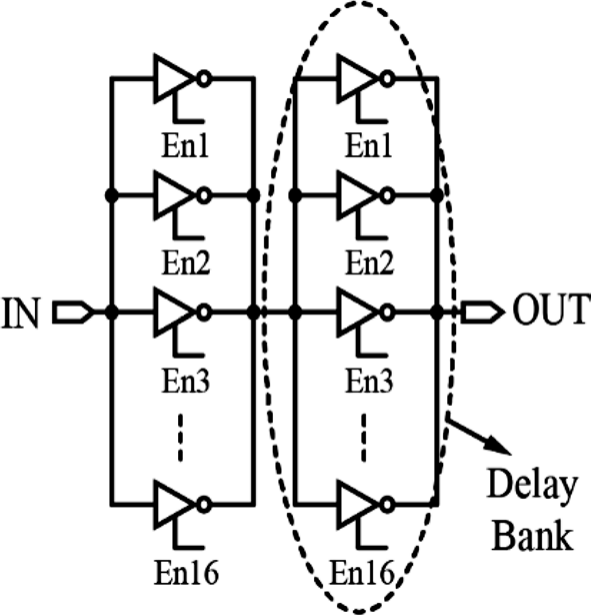
2 × 16 Inverter matrix-based delay element (Abas et al. 2007a)

Another implementation that produces small delay steps is the inverter matrix, as shown in Fig. 13. It is composed of an even number of parallel tri-state inverter banks. The delay of the circuit is adjusted by switching in and out the required number of inverters from the bank (Abas et al. 2007b). This configuration implemented in 0.18 µm CMOS technology can provide fine linear 2 ps delay steps in the output delay region ranging from 84 to 200 ps. The delay range is approximately 400 ps (Abas et al. 2007a).

**Fig. 14:**
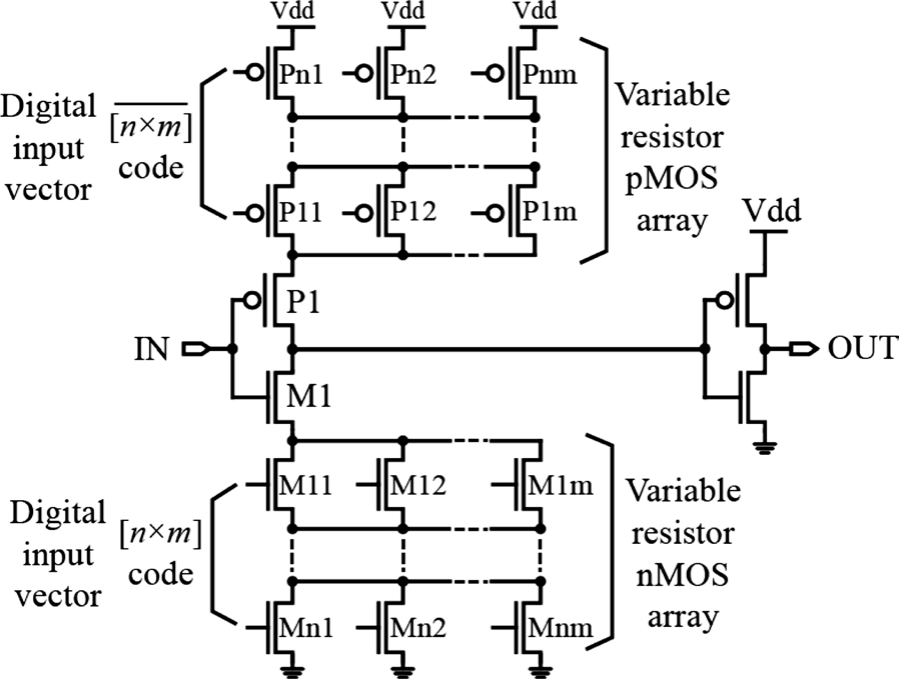
A differential (variable-resistor array) delay element (Saint-Laurent and Swaminathan 2001)

The DDC, sometimes referred to as the variable-resistor array-based delay cell, is shown in Fig. 14.

It consists of two [*n* × *m*] arrays of delay-controlling MOS transistors placed at the source of M1 and P1. Similar to the SCI implementation, the delay-controlling transistors are used to control the output rise and fall times via digital inputs applied to the gates of these transistors that form the variable-resistor array. For example, by turning OFF the transistors, the effective resistance of the variable-resistor array is increased, resulting in increased time delay. The opposite is true when the transistors are turned ON (Saint-Laurent and Swaminathan 2001). Although this seems simple, obtaining perfectly linear delay steps is difficult as the variable resistor design must take into consideration the transistors’ complex parasitic capacitance that changes when the array is turned ON and OFF. This change in the parasitic capacitance can lead to non-monotonic delay behavior with ascending binary pattern of the digital input vector (Maymandi-Nejad and Sachdev 2005). In another implementation of this type of delay elements, the maximum achievable delay resolution was reported to be 1 ps with a delay range of 50 ps implemented in 0.18 μm CMOS technology (Saint-Laurent and Swaminathan 2001).

**Fig. 15:**
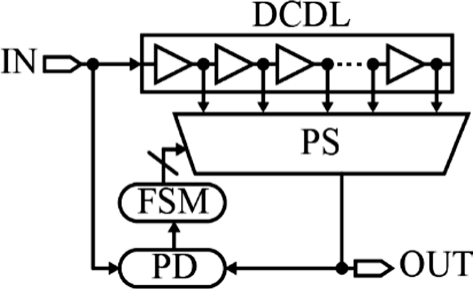
Digital delay-locked loop architecture (Jovanovic et al. 2005)

Likewise, as mentioned in the previous section, phase interpolation can also be implemented by utilizing digital DLLs as delay lines. A sub-gate delay resolution can be achieved by the digital DLLs (Eto et al. 2000; Xanthopoulos 2009). However, the achievable delay resolution of the digital DLLs is not as fine as that of the analog DLLs (Xanthopoulos 2009; Jia 2005). A digital DLL has the advantages of having simpler and more robust design, shorter design time, requiring lower supply voltages (therefore, having a significant reduction in power consumption), having wider range of delay regulation, and enabling better process portability (Jia 2005; Jovanovic et al. 2005; Hsiang-Hui and Shen-Iuan 2005). A digital DLL circuit is shown in Fig. 15. It may include a PD, an up/down counter; a shift register or a finite state machine (FSM), a phase selector (PS), and a DCDL (Jia 2005; Xanthopoulos 2009).

The main difference between analog and digital DLLs is the locking system, or sometimes called the control module as it is responsible for controlling the delay of the delay line based on the output of the PD. In digital DLLs, the locking system can be implemented as a counter, a FSM, or a shift-register. However, in analog DLLs, a CMOS CP with an analog integrated filter is used to implement the locking system (Nuyts et al. 2014; Xanthopoulos 2009). Moreover, analog DLLs generally have smaller footprints as well as better delay resolution, linearity, and jitter performance than digital DLLs.

It is summarized that, like analog-tunable delay elements, digitally-controlled delay elements can also produce high-resolution delay steps according to the current ratio relationship of Eq. (3). However, the main challenge with these delay elements is in realizing uniform linear delay steps/increments. This is because of the change in the complex parasitic capacitance of the digitally-controlled transistors responsible for delay tuning when these transistors are turned ON and OFF. This challenge is mainly noticed with the variable-resistor array delay element (Zhang and Kaneko 2015; Maymandi-Nejad and Sachdev 2005). Nonetheless, this drawback may not be applied provided that proper design techniques are utilized, as in the case with the digitally-controlled SCI delay element implemented by (Miao et al. 2015) which has shown to produce fine and linear delay steps within a relatively wide delay range. The digitally-controlled delay elements have the upper hand when good process portability, short design time, simple and robust design, and good power management are considered together in the design.

## Effects of CMOS technology scaling on delay lines

CMOS technology scaling has shown to improve the delay resolution of the CMOS delay lines. This is clearly recognized in TDLs as the delay resolution of these types of CMOS delay lines relies on the propagation delay of the logic-gates based delay elements. Another positive effect of CMOS technology scaling is the reduction in the power consumption and occupied active area of the fabricated CMOS delay lines chips (Zhang and Kaneko 2015).

On the other hand, degradation of the jitter performance can be the main penalty of the CMOS technology scaling. This is attributed to the noticeably increasing effects of interconnect resistance, NBTI, random doping fluctuations, time-dependent dielectric breakdown (TDDB), hot-carrier injection (HCI) degradation, gate-oxide tunneling, PVT variations, physical-level changes, and short channel effects (Jiang 2011; Segura et al. 2006; Ghahroodi 2014). Therefore, new design techniques should be proposed to overcome the potential excessive jitter when choosing CMOS technologies in the DSM or UDSM nodes.

The following sub-sections address two main areas affecting delay resolution with regards to CMOS technology scaling.

### CMOS technology scaling and delay resolution

The reduction in threshold voltage as CMOS technology scales down is the main factor allowing a finer delay resolution for CMOS delay lines. As a simple observation, according to Eq. (2), as a transistor’s gate length is scaled down, a reduction in the threshold voltage occurs resulting in smaller gate delays. On the other hand, Eq. (5) is used to describe the propagation delay of a CMOS inverter (Sakurai and Newton 1990; Mansour and Shanbhag 2002).

5 $$\tau_{D} = \left( {\frac{{\frac{{V_{TH} }}{{V_{DD} }} + \alpha }}{1 + \alpha } - \frac{1}{2}} \right)t_{T} + \frac{{C_{L} V_{DD} }}{{2I_{D} }} .$$

where *t*_*T*_ and *I*_*D*_ are the input waveform transition time and the drain current with *V*_*GS*_ = *V*_*DD*_, respectively. In Eq. (5), it is obvious that the drain current *I*_*D*_ is directly affecting the gate delay through an inverse relationship. Therefore, it is recommended to investigate the main parameters which are directly influencing *I*_*D*_. This is clearly explained later in this sub-section.

Although it seems that only the change in threshold voltage effects delay as different CMOS technology is used, there are other factors that relate technology scaling to delay resolution and they all are linked with the change in threshold voltage. These factors include the change in oxide thickness and dopant density as we migrate between different CMOS technologies.

For example, a decrease in *T*_*ox*_ causes an increase in gate oxide capacitance, *C*_*ox*_. This is clearly explained by the following equation (Segura and Hawkins 2005; Rabaey et al. 2003):

6 $$C_{ox} = \frac{{\varepsilon_{ox} }}{{T_{ox} }} .$$

where *ε*_*ox*_ is the oxide permittivity.

Furthermore, the increase in *C*_*ox*_ implies an increase in the drain saturation current *I*_*Dsat*_ as illustrated in the following relationship (Segura and Hawkins 2005; Rabaey et al. 2003):

7 $$I_{Dsat} = \frac{{\mu C_{ox} }}{2}\frac{W}{L}\left( {V_{GS} - V_{TH} } \right)^{\alpha }$$

where *V*_*GS*_ is the gate-source voltage. The term (*µC*_*ox*_/2) in Eq. (7) is sometimes referred to it by the symbol κ, which is a coefficient called the process transconductance parameter used to indicate the drive strength of the transistor.

According to Eq. (5), the increase of the drain current in Eq. (7) leads to a decrease in the time delay *τ*_*D*_ of the CMOS inverter gate.

On the other hand, as CMOS technology scales down, the dopant density becomes lower (Segura et al. 2006; Ghahroodi 2014). The mobility of dopant atoms, both nMOS electrons and pMOS holes, directly affects the threshold voltage. According to Eqs. (8a) and (8b) (Kai et al. 1997), the change in the carrier mobility changes the threshold voltage in an inverse relationship (Kai et al. 1997; Akers 1980; Weste and Harris 2011b).

8a $$\mu_{n} = \frac{540}{{1 + \left( {\frac{{V_{GS} + V_{TH} }}{{5.4T_{ox} }}} \right)^{1.85} }},$$

8b $$\mu_{p} = \frac{185}{{1 + \frac{{\left| {V_{GS} + V_{TH} } \right|}}{{3.375T_{ox} }}}}$$

where *μ*_*n*_ and *μ*_*p*_ are the carrier mobility for nMOS electrons and for pMOS holes, respectively. This again affects the threshold voltage in line with what has been discussed for Eq. (5) (Segura et al. 2006; Ghahroodi 2014). It can be concluded that as CMOS technology scales down, the gate delay also decreases as a result of the decrease in threshold voltage due mainly to the change in oxide thickness and carrier mobility.

### Effects of interconnect scaling on delay resolution

As CMOS technology features smaller transistors and lower power supply voltage, interconnecting metal wires also become thinner. In relations to delay line design, this causes the undesired increase in interconnect resistance which ultimately affects the total gate delay. The time constant, τ, of a gate interconnect is given by:

9 $$\tau = \ln \,(2)R_{w} C$$

where *R*_*w*_ is the interconnect resistance and is calculated as:

10 $$R_{w} = \frac{\rho }{w \times t} \times l$$

**Fig. 16:**
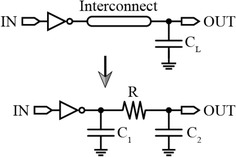
RC–π model of interconnect loads (O’Brien and Savarino 1989)

The resistivity, width, thickness and length of the wire are given as *ρ, w, t* and *l,* respectively. The interconnect width and thickness are inversely proportional with the interconnect resistance. Thus, for a fixed interconnect length, the interconnect resistance increases as *w* and *t* decreases. According to Eq. (10), for long connecting wires, the effects of metal layer-induced capacitance will increase the time constant, which in turn will directly modify the gate delay (Jiang 2011). A one-segment RC–π model presented by O’Brien and Savarino models the interconnect load of a CMOS gate. According to this model, the effect of the interconnect resistance is considered in the gate delay calculation, and the gate’s general RC tree load (an arbitrary RC load) is minimized to three terms, R, C_1_, and C_2_. This network model is shown in Fig. 16. C_1_ and C_2_ is the gate and interconnect capacitances, respectively, and their summation represents the RC-π model’s total capacitance (O’Brien and Savarino 1989). In DSM processes, this resistance reaches several hundreds of ohms.

**Fig. 17:**
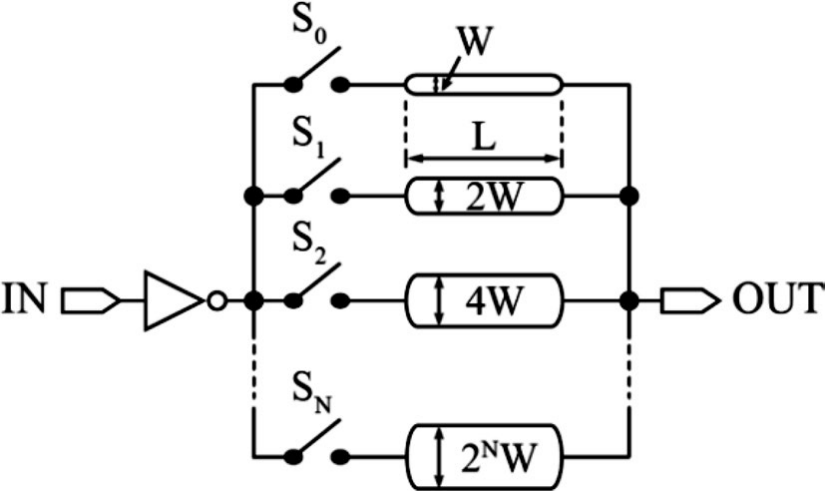
Interconnect array of various binary-weighted interconnect widths

Accordingly, it is concluded that interconnect length and width play a main role in determining the gate delay of digital circuits. This opens the possibility of designing interconnect arrays of various sizes to allow high-resolution delay step for CMOS delay lines, as shown in Fig. 17.

To explain this, the input signal passing through the input inverter is connected to OUT through metal wires with different binary-weighted widths $({\text{W}},2{\text{W}},4{\text{W}}, \ldots ,2^{\text{N}} {\text{W}}).$ These binary-weighted widths act as binary variable resistances which can be activated through CMOS switches (S_0_, S_1_, S_2_, and S_N_). For example, if S_1_ is activated, *R*_*w*_ is reduced to a half of its value compared to when S_0_ is activated. Accordingly, the time constant, τ, is also reduced to a half of its value. The layout of these binary-weighted wires should be considered during the design process to avoid the effects of coupling. Activating two adjacent switches results in forming two adjacent parallel wires, which may in turn lead to unwanted signal coupling effects that will degrade the delay resolution. Signal coupling results in fluctuation of *R*_*w*_ which ultimately changes the time constant value. Another quantity that must be considered in the design is thermal noise whose RMS voltage equation is given by:

11 $$V_{thermal,rms} = \sqrt {4K_{B} TR\Delta f}$$

where *K*_*B*_, *T*, *R*, and ∆*f* are the Boltzmann constant, the absolute temperature in Kelvin, the resistance of the binary-weighted width, and the bandwidth, respectively (Razavi 2001). At high operating frequencies and according to Eq. (11), the thermal noise is increased, which in turn increases the timing jitter.

## Process and environmental (PVT) variations effects on delay line performance

In deep sub-micrometer digital circuits, the delay of both logic gates and interconnects is increasingly affected by parameter (process and environmental) variations and noise sources. Process variations are mainly due to some factors: microscopic non-uniformities in the structure of a circuit during the fabrication process, non-idealities in the nature of the CMOS production process where increasing number of random uncertainties during doping results in fluctuating doping densities and circuit structure defects, increased challenges in controlling the manufacturing process precisely, and limitations in tolerance levels in the lithography stage as the same light source is being used for both, above 130 nm and below, technologies (Ghahroodi 2014; Orshansky et al. 2008). On the other hand, environmental variations occur during the operation of a circuit and are caused by power supply voltage fluctuations and temperature variations that modify the characteristics of transistors in a circuit (Orshansky et al. 2008; Alioto and Palumbo 2006; Segura et al. 2006; Weste and Harris 2011c).

The aforementioned process, supply voltage, and temperature variations are often referred to as PVT variations. The proceeding sub-sections discuss the effects of these variations on CMOS delay line performance.

### Process variations

Process variations are subdivided into inter-die and intra-die variations (Segura et al. 2006; Nuyts et al. 2014; Weste and Harris 2011c). In inter-die/global variations, each device within the same chip will be affected in the same manner as these variations result in an equal random shift in the average value of every device parameter. Process gradients over the wafer (Eisele et al. 1997), variations in the gate oxide thickness (T_ox_) and variations in the exposure time which cause variations in the length and width of transistors are examples of inter-die variations (Henzler 2010a; Alioto et al. 2010). These three inter-die variations examples affect both transistor types, nMOS and pMOS transistors. However, other inter-die variations such as dose variations of ion implantation affect only nMOS or pMOS transistor (Henzler 2010a; Nuyts et al. 2014). During design, these variations are modeled using the well-known process corners, namely, slow–slow (SS), slow–fast (SF), fast–slow (FS), and fast–fast (FF) as they simulate the speed at which CMOS transistors operate. The variations in transistor speed cause the delays for nMOS and pMOS transistors to be different, causing transient fluctuations at the output known as jitter. The jitter level should be taken into consideration as it directly affects delay resolution (Nuyts et al. 2014).

**Fig. 18:**
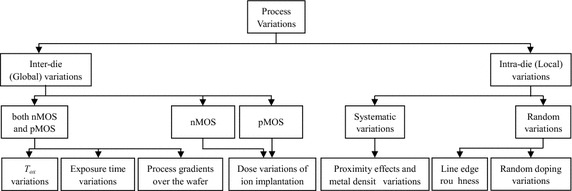
A classification of the main process variations found in CMOS delay lines

On the other hand, intra-die variations, also called local variations, cause different devices within the same chip to have different properties. Statistical variations of doping concentrations, line edge roughness (Henzler 2010a) and proximity effects are examples of intra-die variations (Alioto et al. 2010; Eisele et al. 1997). In DSM and UDSM CMOS technologies, these three examples of intra-die variations become more noticeable. To illustrate this, the random variations in the threshold voltage are significantly increased as the presence or absence of a single atom of the dopant atoms will have a more significant effect on the overall device performance compared with large-scale CMOS technologies (Ghahroodi 2014). For transistors located closely to each other, these variations are usually correlated (Nuyts et al. 2014). In addition, in DSM and UDSM CMOS technologies, these variations are also classified into two categories, random and systematic variations. Random intra-die variations, such as random doping variations, impact devices such as transistors and interconnects in a different way even in the case they are relatively close. Systematic variations, such as proximity effects and metal density variations, impact close devices (transistors) in the same way (Alioto et al. 2010; Ghahroodi 2014). These local process variations lead to device mismatch, which in turn leads to degraded delay resolution due to excessive jitter. The main process variations for CMOS delay lines are summarized in Fig. 18.

**Fig. 19:**
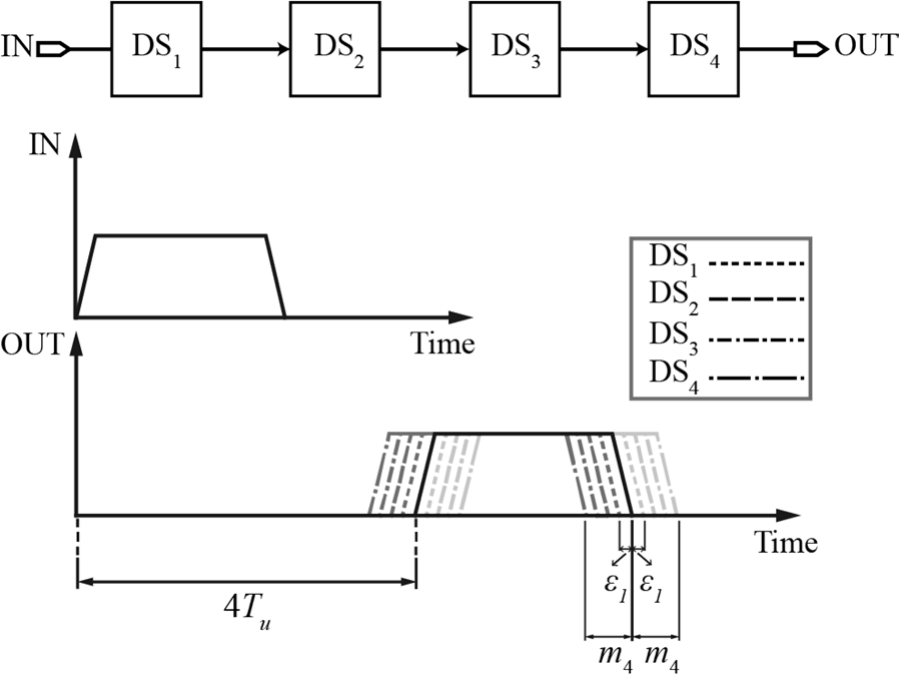
Jitter due to intra-die/local process variations

Figure 19 shows the effect of intra-die variations on the generated time delay of a CMOS delay line composed of four cascaded identical delay stages, where DS stands for a delay stage. The output drawn in solid line is the ideally delayed output of 4*T*_*u*_. However, the output drawn in dotted lines is delayed by 4*T*_*u*_ plus the delay uncertainty introduced by subsequent delay stages. In other words, Fig. 19 shows the delay uncertainty of the CMOS delay line represented by the accumulation of timing jitter along the delay path.

The delay error, *ε*, which can be positive or negative, affects the time delay of each stage along the delay line and is described by the following equation (Henzler 2010a):

12 $$t_{d,n} = n \cdot T_{u} + \sum\limits_{i = 1}^{n} {\varepsilon_{i} }$$

where *T*_*u*_, *t*_*d*,*n*_, and *n* are the absolute resolution, actual delay of the *n*th delay stage, and the number of a specific delay element in the delay line, respectively. In Fig. 19, *m*_4_ refers to the total accumulated delay errors at the end of the delay line. For a multiple-stage delay line, the delay deviations and uncertainties are strongly correlated, meaning that they accumulate along the delay stages. In the case that the delays of the stages vary independently, the standard deviation is written as (Nuyts et al. 2014; Henzler 2010a):

13 $$std(\varepsilon_{i} ) = std(\varepsilon ).$$

The delay uncertainty at the end of the CMOS delay line is given by:

14 $$std(t_{d,N} ) = std(\varepsilon ) \cdot \sqrt N .$$

Equation (14) shows that the time delay variation of stage *N*, *std(t*_*d,N*_*)*, is equal to the time delay error multiplied by the square root of the number of delay stages.

Device mismatch, which is caused by intra-die/local variations as mentioned earlier, in delay elements causes delay uncertainty or jitter which in turn degrades the *differential non*-*linearity* (*DNL*) and *integral non*-*linearity* (*INL*) of the delay line. *DNL* and *INL* are two performance measures of CMOS delay lines similar to DACs (Nuyts et al. 2014). They are quantities for measuring systematic errors that cause the delay increments to differ from their ideal values (Li 2010; Jansson et al. 2005; Rahkonen and Kostamovaara 1993). *DNL* can be defined as the delay deviation of the *i*th delay step from its ideal value *T*_*u*_. *DNL* indicates the precision of a delay line output according to its input code. On the other hand, *INL* is defined as the deviation of the *n*th delay step position from its normalized ideal value determined by a straight line connecting the first and the last steps. It specifies the linearity of the overall delay line (Li 2010; Nuyts et al. 2014; Henzler 2010a). Many high-speed and high-performance CMOS VLSI circuits impose strict linearity requirements represented by the achievement of highly-monotonic and linear delay steps by the designed CMOS delay line for the entire attainable delay range (Sakamoto et al. 1989; Maymandi-Nejad and Sachdev 2005). Hence, *DNL* can directly be obtained by the delay variation of a particular delay element as (Henzler 2010a):

15 $$DNL_{i} = \frac{{(t_{d,i + 1} - t_{d,i} ) - T_{u} }}{{T_{u} }} = \frac{{\varepsilon_{i + 1} }}{{T_{u} }}.$$

For the calculation of the *INL*, the reference delay step position, $t_{d,n}^{\prime } ,$ is first calculated for each step as follows (Henzler 2010a):

16 $$t_{d,n}^{\prime } = t_{d,1} + \frac{n - 1}{N - 1}(t_{d,N} - t_{d,1} ).$$

Then, the *INL* at a specific *n*th delay step is calculated in terms of *ε*_*i*_ as follows (Henzler 2010a; Nuyts et al. 2014):

17 $$INL_{n} = \frac{{t_{d,n} - t_{d,n}^{\prime } }}{{T_{u} }} = \frac{1}{{T_{u} }}\left( {\sum\limits_{i = 2}^{n} {\varepsilon_{i} \left[ {1 - \frac{n - 1}{N - 1}} \right]} - \frac{n - 1}{N - 1}\sum\limits_{i = n + 1}^{N} {\varepsilon_{i} } } \right).$$

Alternatively, since the *INL* represents the cumulative sum of the *DNL*, the *INL* can also be calculated as follows (Nuyts et al. 2014):

18 $$INL_{n} = \sum\limits_{i = 1}^{n} {DNL_{i} } = \frac{{\sum\nolimits_{i = 1}^{n} {\left[ {t_{d,i + 1} - t_{d,i} } \right] - nT_{u} } }}{{T_{u} }} = \frac{{\sum\nolimits_{i = 1}^{n} {\varepsilon_{i} } }}{{T_{u} }}.$$

As shown in Eqs. (15), (17) and (18), both the *INL* and *DNL* are normalized to one *T*_*u*_ (Henzler 2010a; Li 2010). On the other hand, when utilizing a DLL as a delay line, the locking system of the DLL forces the delay to be locked to a specific value regardless of any intra-die or inter-die variations. This implies that the total jitter of the delay line is forced to be zero although the delay uncertainty for the individual delay elements is not equal to zero and is given by:

19 $$std(t_{d,n} ) = std(\varepsilon ) \cdot \sqrt {\frac{n \cdot (N - n)}{N}} .$$

Equation (19) has its maximum value at *N*/2 (Nuyts et al. 2014; Henzler 2010a). Moreover, locking the delay lines’ delay eliminates the inter-die/global variations effect and reduces the intra-die/local variations effect (Nuyts et al. 2014).

### Environmental [power supply voltage and temperature (VT)] variations

Environmental variations sources can vary in time and space according to the power consumption, and they highly contribute to the delay uncertainty (Segura et al. 2006; Weste and Harris 2011d; Alioto and Palumbo 2006). These variations have a global effect on the performance of CMOS delay lines (Henzler 2010a). For example, supply voltage fluctuations in the supply distribution network are caused by time-varying voltage drops during switching activity, which are coupled to other circuit blocks through supply/ground wires (Alioto and Palumbo 2006; Segura et al. 2006). This results in increased power supply noise and ultimately increases jitter. The delay sensitivity to supply voltage fluctuations, $S_{{V_{DD} }}^{{\tau_{D} }} ,$ will become larger and more significant in the UDSM technology nodes (Alioto and Palumbo 2006). In practical cases, supply voltage ∆variation, *V*_*DD*_, in the range 5–10 % is tolerated and acceptable in VLSI circuits. This implies that the ratio ∆*V*_*DD*_/*V*_*DD*_ is small; as a consequence, the dependence of delay on the supply voltage variation can be measured via the delay sensitivity equation with regards to the supply voltage (Alioto and Palumbo 2006):

20 $$S_{{V_{DD} }}^{{\tau_{D} }} = \mathop {\lim }\limits_{{\Delta V_{DD} \to 0}} \frac{{\frac{{\Delta \tau_{D} }}{{\tau_{D} }}}}{{\frac{{\Delta V_{DD} }}{{V_{DD} }}}} = \frac{{V_{DD} }}{{\tau_{D} }} \cdot \frac{{d\tau_{D} }}{{dV_{DD} }}.$$

On the other hand, the fluctuation in the output time delay due to temperature variations is attributed to two processes: threshold voltage variation and carrier mobility fluctuation (Segura et al. 2006; Kumar and Kursun 2006). The threshold voltage magnitude is reduced as temperature increases as illustrated in the following equation (Weste and Harris 2011b):

21 $$V_{TH} (T) = V_{TH} (T_{r} ) - k_{vt} (T - T_{r} )$$

where *T*, *T*_*r*_, and *k*_*vt*_ are the absolute temperature, the room temperature, and a constant whose value is typically about 1–2 mV/K, respectively.

According to Eq. (2), the reduction in the threshold voltage leads to an improvement in the gate delay. The threshold voltage reduction is accompanied by a relative increase in drain saturation current due to the increase in gate overdrive voltage, *V*_*GS*_–*V*_*TH*_ (Segura et al. 2006; Kumar and Kursun 2006). This also implies a reduction in the gate delay according to Eq. (5).

On the other hand, the relationship between the carrier mobility and the temperature is formulated as follows (Weste and Harris 2011b):

22 $$\mu (T) = \mu (T_{r} )\left( {\frac{T}{{T_{r} }}} \right)^{{ - k_{\mu } }}$$

where *T*, *T*_*r*_, and *k*_*µ*_ are the absolute temperature, the room temperature, and a fitting parameter whose value is typically about 1.5.

It is obvious from Eq. (22) that the carrier mobility is inversely proportional to temperature. For example, when temperature increases, the mobility is decreased, resulting in slow switching of the transistors of the delay elements. Hence, the propagation delay is increased and vice versa (Kumar and Kursun 2006; Segura et al. 2006; Weste and Harris 2011c; Cheng and Milor 2009; Rahkonen and Kostamovaara 1993).

For the aforementioned description about the PVT variations, it can be summarized that these variations highly affect the jitter performance and delay resolution of the CMOS delay line. Therefore, it should be noted that the total delay fluctuations due to these variations are required to be less than the delay resolution in many applications especially for the high-resolution and high-frequency applications (Zhang and Kaneko 2015).

## Delay line noise and jitter

**Fig. 20:**
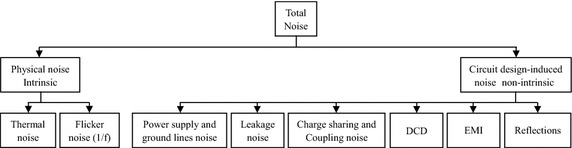
A classification of the main noise sources found in CMOS delay lines

In CMOS delay lines, the deviation of the output pulse amplitude is known as amplitude noise and the deviation of the output time delay is known as timing jitter. The noise sources are classified into two: physical noise sources and circuit design-induced noise sources. Physical noise, also called intrinsic noise, is caused by the random fluctuation and stochastic nature of electronic charge carriers implanted in the device during fabrication. It may be reduced but cannot be eliminated completely (Li 2008; Figueiredo and Aguiar 2006; Henzler 2010a). It is the main source of jitter in delay lines. The most common types of physical noise are thermal, flicker (1/f), and shot noise (Li 2008; Figueiredo and Aguiar 2006). For delay elements’ MOS transistors, the noise of these devices mainly involves thermal and flicker noise (Cheng and Milor 2009). Physical noise causes delay shifts in the output signal of the CMOS delay line. The effect of these delay shifts on the delay step is explained by assuming that each delay element contributes to a certain noise error-induced delay shift which accumulates along the delay line (Henzler 2010a):

23 $$t_{d,N} = N \cdot T_{u} + \sum\limits_{i = 1}^{N} {\eta_{i} }$$

where *t*_*d*,*N*_, *N*, *T*_*u*_, and *η* are the actual time delay at the end of the CMOS delay line, the total number of the delay elements in the delay line, the absolute resolution, and the delay shift which is induced by noise error, respectively. On the other hand, circuit design-induced noise, also called non-intrinsic noise, is due to circuit switching activities that cause fluctuations in currents and voltages. This noise can be minimized and even removed if careful design techniques are utilized. This noise can be classified into many subclasses, namely, power supply and ground lines noise, leakage noise, charge-sharing and coupling noise, duty-cycle distortion (DCD), electromagnetic interference (EMI), and reflections (Shepard and Narayanan 1996; Figueiredo and Aguiar 2006; Li 2008). All of these noise sources ultimately contribute to timing jitter. The main noise sources of CMOS delay lines are summarized in Fig. 20.

Timing jitter is classified into three types: absolute jitter, cycle jitter, and cycle-to-cycle jitter (Zhang et al. 2004). The absolute jitter ∆*T*_*abs*_, also called long-term jitter, is the accumulated jitter for *N* clock cycles and is given by Zhang et al. (2004):

24 $$\Delta T_{abs} (N) = \sum\limits_{n = 1}^{N} {\Delta T_{n} .}$$

Cycle jitter, ∆*T*_*c*_, represents the long-term average effect of clock cycle fluctuation and can be described by the long-term RMS value of ∆*T*_*n*_ which is the difference between the actual period of the *n*th clock cycle and that of its ideal counterpart. It is written as:

25 $$\Delta T_{c} = \mathop {\lim }\limits_{N \to \infty } \sqrt {\frac{1}{N}\sum\limits_{n = 1}^{N} {\Delta T_{n}^{2} .} }$$

Finally, cycle-to-cycle jitter, ∆*T*_*c*–*c*_, represents the RMS difference between two successive clock cycles and is written as (Cheng and Milor 2009; Zhang et al. 2004):

26 $$\Delta T_{c - c} = \mathop {\lim }\limits_{N \to \infty } \sqrt {\frac{1}{N}\sum\nolimits_{n = 1}^{N} {\left( {T_{n + 1} - T_{n} } \right)^{2} } } .$$

Jitter due to noise can be classified into random jitter and deterministic jitter. Random jitter, also referred to as non-systematic jitter, is an unpredictable jitter component whose amplitude is unbounded and Gaussian in nature. On the other hand, deterministic jitter, also known as systematic jitter, is a predictable jitter component whose amplitude is bounded. Physical noise sources are considered as the major contributor to random jitter in CMOS delay lines. Circuit design-induced noise sources also contribute to random jitter; however, they contribute more to deterministic jitter through DCD, EMI, charge-sharing and coupling noise. Device mismatch caused by intra-die process variations also contributes to deterministic jitter (Jia 2005; Li 2008).

The influence of noise on jitter is strongly related to the output load capacitance and the short-circuit current of the delay line. The total jitter of a delay line can be obtained by the sum of variances of the time delay produced by each delay stage if the noise sources are uncorrelated. However, these jitter components are correlated through the power supply rails and their respective noise components. Taking this into consideration, the total jitter is higher because of the correlation effect (Figueiredo and Aguiar 2006).

**Fig. 21:**
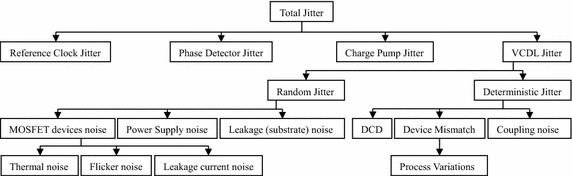
Total Jitter of a conventional analog DLL and its sources

Referring to Fig. 7 and focusing on delay elements of the VCDL, a DLL’s jitter classification with the root main causes can be concluded in Fig. 21.

## Open research issues and conclusion

It has been shown that there is a trade-off between delay resolution and dynamic range for the different types of delay line circuits. In other words, a higher-resolution delay line will have a shorter dynamic range and vice versa. This can be seen clearly by comparing the delay resolution and dynamic range values for different types of delay line circuits, as shown in Table 1. Consequently, this reflects the necessity of developing a CMOS delay line circuit which fulfills each of these delay specifications in one single circuit block. Some suggestions regarding this trade-off challenge are discussed at the end of this section. Table 1 also addresses normalized absolute *DNL* and *INL* values as well as control mechanism type for main types of delay elements implemented using 0.18 μm CMOS technology.

**Table 1:** Delay resolution and delay range for the main delay line types

Delay element	Technology (μm)	Delay resolution (ps)	Delay range	Power consumption	Area	|*DNL*| (normalized)	|*INL*| (normalized)	Jitter (RMS)	Control mechanism
1. Variable-resistor array (Saint-Laurent and Swaminathan 2001)	0.18	1	50 ps	N/A	N/A	1.37	1.04	N/A	Digital
2. Inverter matrix (Abas et al. 2007a)	0.18	2	116 ps	N/A	3465 μm^2^	2.7	3.11	N/A	Digital
3. SCI (Abas et al. 2007a)	0.18	2	32 ps	N/A	1140 μm^2^	0.25	1	N/A	Digital
4. Supply modulation-based (Moazedi et al. 2011)	0.18	30	1.34 ns	0.4 mW	N/A	9	17	9.5 ps	Analog
5. CSI (Seraj et al. 2015)	0.18	400	4 ns	N/A	N/A	0.18	0.26	N/A	Analog
6. CSI (Maymandi-Nejad and Sachdev 2005)	0.18	2	320 ps	0.17–0.34 mW	5000 μm^2^	13.61	36.15	N/A	Digital
7. DLL (Chung-Ting et al. 2009)	0.18	70	1.57 ns	2.4–4.2 mW	0.2584 mm^2^	6.5	6	3.8 ps	Analog

On the other hand, Table 2 focuses on the power consumption and area values for different types of delay elements implemented using 0.35 μm CMOS technology. Delay resolution and range values are also provided in Table 2, illustrating the difference in these two parameters when compared to their 0.18 μm counterparts in Table 1. It should be noted that the values for power consumption and area for all delay elements in Table 2 are provided by (El Mourabit et al. 2012) via simulating the circuits using 0.35 μm CMOS technology. For the CSI in Table 2, all the provided parameters values are extracted by (El Mourabit et al. 2012).

**Table 2:** Power consumption and area for different delay line types

Delay element	Technology (μm)	Delay resolution (ps)	Delay range (ps)	Power consumption	Area	|*DNL*| (normalized)	|*INL*| (normalized)	Jitter (RMS)	Control mechanism
1. CMOS inverter based on RC differentiator (El Mourabit et al. 2012)	0.35	0.5	200	100 μW @ 0.5 GHz	400 μm^2^	0.4	0.1	N/A	Digital
2. SCI (Pao-Lung et al. 2005)	0.35	1.43	40	490 μW @ 400 MHz	850 μm^2^	0.37	0.19	N/A	Digital
3. CSI (Maymandi-Nejad and Sachdev 2003)	0.35 (El Mourabit et al. 2012)	40	400	211 μW @ 400 MHz	450 μm^2^	–	–	N/A	Digital
4. Cascaded inverters (Ching-Che and Chen-Yi 2003)	0.35	5	300	950 μW @ 400 MHz	0.36 mm^2^	–	–	N/A	Digital

Although analog-tunable delay elements with high-resolution delay steps may not be shown clearly in Table 1, they have the upper hand in terms of achieving higher-resolution delay steps because of the fact that the delay is being controlled precisely by the current ratio relationship according to Eq. (3). Moreover, analog-tunable delay elements have lower jitter and better intrinsic calibration for PVT variations (Markovic et al. 2013).

Fine delay control according to current ratio relationship of Eq. (3) can also be attained with digitally-controlled delay elements. However, this might be achieved but at the cost of linearity degradation as the complex parasitic capacitance of the digitally-controlled transistors changes when the transistors are turned ON and OFF (Maymandi-Nejad and Sachdev 2005). Furthermore, the jitter performance of the digitally-controlled delay elements is not as fine as that of the analog-tunable delay elements. This is because that for a high-resolution digital delay line, if the controller always switches the control code of the delay line, the jitter performance will not be good. Alternatively, digitally-controlled delay elements have wider range as well as simpler control of delay regulation, simpler and more robust design, lower power consumption, and better process portability than analog-tunable delay elements.

Programmable delay lines with sub-gate delay resolution have been realized using many circuit topologies and techniques. They mainly involve: changing the capacitive loading (SCI) mechanism reported in (Schidl et al. 2012; Abas et al. 2007a; Pao-Lung et al. 2005; Miao et al. 2015) and some of the current-starving delay-controlling techniques reported in (Maymandi-Nejad and Sachdev 2005; Saint-Laurent and Swaminathan 2001; El Mourabit et al. 2012) which are mentioned earlier in the third and fourth sections of this paper, delay difference between two delay paths (sometimes called VDL) (Xanthopoulos 2009; Guang-Kaai et al. 2000; Nuyts et al. 2014), employing phase interpolation technique through, for example, utilizing DLLs as delay lines (Xanthopoulos 2009; Yang 2003), utilizing a capacitor charging along with comparators controlled by a DAC (Suchenek 2009; Klepacki et al. 2015), and utilizing an analog differential buffer (Nuyts et al. 2014).

As illustrated in this study, the jitter performance is dependent on the PVT variations and noise sources of the delay line circuit. However, another important factor that affects the jitter performance is how the CMOS delay line is controlled, i.e. analog or digital control mechanism as illustrated earlier.

The delay-controlling/tuning techniques of the delay elements reported in this paper are mainly based on changing the drive strength of the delay element, except for the SCI and MOS-diode-based delay elements in which the delay tuning is based on load-increasing strategy. Accordingly, a comparison for most of the design specifications is presented in Table 3 for three different delay elements which are: CSI, as an example in which the delay is tuned by changing the drive strength, the SCI and the Inverter Chain. These delay elements are all digitally-controlled and implemented using 0.18 μm CMOS technology (Zhang and Kaneko 2015). Referring to Table 3, the range of the sequence from 1 to 3 corresponds to the performance of each delay element with regard to each design specification. To illustrate this, sequence number 1 refers to the best case, number 3 to the worst case, and number 2 to in between case. The following descriptions compare and discuss the differences in the performance among these delay elements with respect to the design specifications starting with delay resolution and ending with robustness against temperature variation.

**Table 3:** Comparison of main design specifications for CSI, SCI and inverter chain delay elements

S. no.	Delay resolution	Delay range	Power consumption	Area	Linearity	Robustness against process variation	Robustness against temperature variation
1	SCI	Inv. Chain	CSI	CSI	Inv. chain	Inv. chain	SCI
2	CSI	SCI	SCI	SCI	SCI	SCI	CSI
3	Inv. chain	CSI	Inv. chain	Inv. chain	CSI	CSI	Inv. chain

To start with, the resolution of the inverter chain is dependent on the CMOS process, thereby limiting the maximum achievable resolution of inverter-based buffer to approximately tens of picoseconds. The delay resolution of the CSI is not as fine as that of the SCI because of the delay-controlling transistors placed at the discharging and/or charging paths that limit the amount of the load capacitor’s (dis)charging current. Accordingly, the delay range of the CSI is shorter than that of the SCI (Zhang and Kaneko 2015).

For a specific required delay range, a large number of redundant inverters is required by the Inverter Chain and hence, higher power is consumed and larger area is occupied. The power consumption and the area are increased for the SCI when the delay increases since a heavy load capacitance is needed. However, a lower power is consumed when a larger delay is required for the CSI (Zhang and Kaneko 2015).

The effect of process variations on the delay steps between delay stages of the Inverter Chain is considered small compared to the relatively large delay step of the inverter-based buffer. Thus, the Inverter Chain achieves better linearity compared to the SCI and CSI in which the minimum attainable delay step is much smaller than that of the inverter chain (Zhang and Kaneko 2015). When both discharging and charging paths of the CSI contain digitally-controlled transistors, the effect of complex parasitic capacitance interaction becomes more noticeable and leads to more delay fluctuations than the SCI especially when the delay increases. Moreover, unlike the CSI, the current of the SCI does not decrease when the delay increases. This makes the SCI more robust against variations than the CSI (Zhang and Kaneko 2015).

The inverter chain has a poor robustness against temperature changes as the resulted delay fluctuations accumulate along the delay line in a correlation relation. Likewise, the resulted delay fluctuations of the CSI accumulate along each transistor in the discharging/charging paths. However, a heavy load can be utilized for the SCI to enhance the robustness against the temperature variations but at the cost of increased power consumption and area (Zhang and Kaneko 2015).

Back to the trade-off challenge between delay resolution and delay range, some solutions are being proposed in order to overcome this challenge. Cascading multiple delay lines with different specifications is one of the possible solutions (Xanthopoulos et al. 2001; Xanthopoulos 2009). For example, a coarse counter is cascaded with interpolators based on digital delay lines (Kalisz 2004). Another solution is to employ both analog and DCDLs together in one single design to make use of the unique advantages of both of these differently controlled CMOS delay line types (Markovic et al. 2013; Miao et al. 2015). Utilizing phase interpolators with DCDLs is another solution without the need for cascading multiple DCDLs (Xanthopoulos 2009). A combination of integrated CMOS delay lines technique with other time-interval generation techniques as in Klepacki et al. (2014, 2015), Suchenek (2009) can also be another solution.

It should be mentioned that the benefits gained from utilizing these possible solutions are achieved at the cost of potential increasing in power consumption, occupied area, jitter, non-uniform linearity, and control complexity. Depending on the requirements of the applications in which these delay lines are employed, these shortcomings may be acceptable and compromised in favor of achieving both high delay resolution and wide delay range.
